# A temperate *Siphoviridae* bacteriophage isolate from Siberian tiger enhances the virulence of methicillin-resistant *Staphylococcus aureus* through distinct mechanisms

**DOI:** 10.1080/21505594.2021.2022276

**Published:** 2022-01-06

**Authors:** Dan Yang, Shuang Wang, Erchao Sun, Yibao Chen, Lin Hua, Xiangru Wang, Rui Zhou, Huanchun Chen, Zhong Peng, Bin Wu

**Affiliations:** a State Key Laboratory of Agricultural Microbiology, College of Veterinary Microbiology, Huazhong Agricultural University, Wuhan, China; b Ministry of Agriculture and Rural Affairs Key Laboratory of Development of Veterinary Diagnostic Products, the Cooperative Innovation Center for Sustainable Pig Production, Huazhong Agricultural University, Wuhan, China; c Ministry of Science and Technology International Research Center for Animal Disease, Huazhong Agricultural University, Wuhan, China

**Keywords:** Bacteriophage, methicillin-resistant *Staphylococcus aureus*, biofilm formation, virulence, lysogens

## Abstract

The emergence and worldwide spread of Methicillin-resistant *Staphylococcus aureus* (MRSA) pose a threat to human health. While bacteriophages are recognized as an effective alternative to treat infections caused by drug resistant pathogens, some bacteriophages in particular the temperate bacteriophage may also influence the virulence of the host bacteria in distinct ways. In this study, we isolated a bacteriophage vB_Saus_PHB21 from an epidermal sample of Siberian tiger (*Panthera tigris altaica*) using an MRSA strain SA14 as the indicator. Our following laboratory tests and whole genome sequencing analyses revealed that vB_Saus_PHB21 was a temperate bacteriophage belonging to the *Siphoviridae* family, and this bacteriophage did not contain any virulence genes. However, the integration of PHB21 genome into the host MRSA increased the bacterial capacities of cell adhesion, anti-phagocytosis, and biofilm formation. Challenge of the lysogenic strain (SA14^+^) caused severe mortalities in both *Galleria mellonella* and mouse models. Mice challenged with SA14^+^ showed more serious organ lesions and produced higher inflammatory cytokines (IL-8, IFN-γ and TNF-α) compared to those challenged with SA14. In mechanism, we found the integration of PHB21 genome caused the upregulated expression of many genes encoding products involved in bacterial biofilm formation, adherence to host cells, anti-phagocytosis, and virulence. This study may provide novel knowledge of “bacteria-phage-interactions” in MRSA.

## Introduction

The rapid emergence and dissemination of drug-resistant pathogens have posed a big threat to global public health [1]. One drug-resistant pathogen of great concern is methicillin-resistant *Staphylococcus aureus* (MRSA), which is a cause of staph infections that are difficult to treat because of resistance to some antibiotics. Since its first description in 1960s, MRSA has become a leading cause of bacterial infections in both health-care and community settings [2]. Compared to the infections due to methicillin-susceptible *S. aureus*, MRSA causes infections with higher mortality rates and results in increased lengths of hospital stays as well as associated health care costs [3]. In recent years, even with the ongoing development of new antibiotics, active surveillance efforts and advances in infection prevention, MRSA remains a prominent pathogen with persistently high mortality [4]. In 2017, the World Health Organization (WHO) listed MRSA as a pathogen of high priority that urgently requires new therapeutic options [5]. In addition to humans, MRSA strains can also persist and cause infections in many animals, including domestic and wild animals [6]

Known as the natural predators of bacteria, bacteriophages (thereafter referred as phages) have been recognized as a good alternative for treating infections caused by drug resistant pathogens since their discovery in 1915 [7]. Indeed, phage therapy has achieved a great success in combating drug resistant bacterial infection particularly those caused by multidrug resistant pathogens in several cases during the past decades [8,9]. However, phage therapy still faces some problems, of particular concern is the safety [10]. Many phages in particular the temperate phages have been found to help transfer antimicrobial resistance genes (ARGs) and/or virulence factor genes (VFGs), thereby conferring the bacteria antimicrobial resistance and/or increasing bacterial virulence [11,12]. In this study, we isolated a temperate phage that does not carry any VFGs from an epidermal sample of a Siberian tiger (*Panthera tigris altaica*) using a MRSA strain as the indicator. However, we found the integration of this phage genome significantly enhanced the virulence of host MRSA strain. To explore the related mechanism, we performed RNA-Seq analysis and we found the integration of this phage genome caused the upregulated expression of many genes related to the biofilm formation and virulence in the lysogenic MRSA strain. Our findings will provide novel knowledge of “bacteria-phage-interactions” in MRSA.

## Methods

### Bacterial strains, phage isolation and purification, electron microscopy, and host specificity

MSRA strains used in this study included those isolates from both humans and pigs in different regions in China (Table 1). These isolates were kindly gifted by Prof. Rui Zhou at Huazhong Agricultural University, Wuhan, China. A human origin MRSA strain SA14 was used as the indicator for bacteriophage isolation. Bacteriophage was isolated and purified according to methods described previously [13,14]. Briefly, epidermal swabs were suspended in 1 ml of PBS, which was then centrifuged at 2100 × *g* for 10 min. The supernatants were sterilized by filtration through a 0.22 μm pore size membrane (Millex–GP, USA). After that, 300 μl of the filtrates were mixed with 300 μl of the indicator bacterium (MRSA SA14) in exponential growth phase, and was poured on the double-layer plate, incubating at 37°C for 12 h. After the plaques were enumerated, a single plaque was picked and was resuspended in sterile SM buffer (100 mM NaCl, 8.5 mM MgSO_4_ · 7H_2_O, 50 mM Tris-Cl (pH 7.5), and 0.01% gelatin). The phage-containing SM buffer was filtered again through a 0.22 μm pore size membrane, and phage isolation by the double-layer agar method was repeated four more times. Finally, the phages were purified by CsCl gradient ultra-centrifugation [13]. Purified phage suspensions were stored at 4°C for further use.

**Table 1.** Host spectrum of bacteriophage PHB21

Bacterial species	Strains	Hosts	Places of isolation	Sequence types	Plaque formation	EOP
*Staphylococcus aureus*	SA1	Human	Hubei, China	ST968	-	-
	SA2	Human	Hubei, China	ST9	-	-
	SA3	Human	Hubei, China	ST9	+	0.57
	SA4	Human	Hubei, China	ST968	-	-
	SA5	Human	Hubei, China	ST9	-	-
	SA6	Human	Hubei, China	ST9	-	-
	SA7	Human	Hubei, China	ST9	+	0.43
	SA8	Human	Hubei, China	ST9	-	-
	SA9	Human	Hubei, China	ST9	+	0.25
	SA10	Human	Hubei, China	ST9	-	-
	SA-A	Human	Hubei, China	ST9	-	-
	SA12	Human	Hubei, China	ST9	+	0.62
	SA-B	Pig	Hubei, China	ST9	-	-
	SA-C	Pig	Hubei, China	ST9	-	-
	SA15	Pig	Hubei, China	ST9	-	-
	SA16	Pig	Hubei, China	ST9	-	-
	SA17	Pig	Hubei, China	ST9	+	0.12
	SA18	Pig	Hubei, China	ST9	+	0.44
	SA19	Pig	Hubei, China	ST9	-	–
	SA20	Pig	Hubei, China	ST9	+	0.04
	SA21	Pig	Hubei, China	ST9	+	0.08
	SA22	Pig	Hubei, China	Unknown	+	0.16
	SA23	Pig	Hubei, China	ST9	-	-
	SA24	Pig	Hubei, China	ST9	+	0.69
	SA25	Pig	Hubei, China	ST9	-	-
	SA26	Pig	Hubei, China	ST9	-	-
	SA27	Pig	Hubei, China	ST9	-	-
	SA28	Pig	Hubei, China	ST1376	+	0.003
	SA29	Pig	Hubei, China	ST9	+	0.046
	SA30	Pig	Hubei, China	ST1376	+	0.57
*Staphylococcus chromogenes*	Hb-1	Pig	Hubei, China	Unknown	-	-
	Hb-2	Pig	Hubei, China	Unknown	-	-
*Bordetella bronchiseptica*	D1	Pig	Hubei, China	Unknown	-	-
	D2	Pig	Hubei, China	Unknown	-	-
*Pasteurella multocida*	6192	Pig	Hubei, China	ST11	-	-
	6305	Pig	Hubei, China	ST10	-	-
	HN07	Pig	Henan, China	ST12	-	-
*Enterococcus faecalis*	EF3964	Human	Hubei, China	Unknown	-	-
*Salmonella* Typhimurium	59	Pig	Hubei, China	Unknown	-	-
*Salmonella* Derby	22	Pig	Hubei, China	Unknown	-	-
*Escherichia coli*	O157	Pig		Unknown	-	-
	DH5α	-		Unknown	-	-

Phage morphology was observed using a 100-kV transmission electron microscope (HITACHI H-7650, Tokyo, Japan) with the same protocol as described by Chen et al. [15]. Bacteriophage host range was determined by spot tests according to methods described previously [13]. In addition to the MRSA strains mentioned above, our laboratory collected bacterial strains belonging to the other species including *P. multocida, B. bronchiseptica, Salmonella, E. faecalis*, and *E. coli* were also used to determine the host specificity of phage isolated (Table 1).

### Generating PHB21 lysogens

The phage isolated herein, designated vB_Saus_PHB21 (thereafter referred as PHB21; 1 × 10^9^ PFU), was spotted on a lawn of MRSA strain SA14 on tryptic soy agar (TSA) plates (Becton, Dickinson and Company, MD, USA) and incubated overnight at 37°C. Bacterial colonies within the lysis zone were isolated and were examined for the sensitivity against PHB21 infection by spot tests. Those (designated SA14^+^) with phenotypes of resistance to phage infection were set for PCR amplification of the integrase gene (CDS28), *cI* gene (CDS32), major capsid protein gene (CDS5) and large terminase subunit (CDS2) to confirm the presence of PHB21 genome in the bacterial chromosome.

### Growth test

Overnight culture of SA14^+^ and/or SA14 was transformed into fresh tryptic soy broth (TSB) medium (Becton, Dickinson and Company, MD, USA) at a ratio of 1:100 (*v*/*v*), which was shaken at 180 rpm, 37°C. The time point when the shaking culture started was set as 0 h, and from this time point, 100 µL bacterial culture was plated on tryptic soy agar (TSA; Becton, Dickinson and Company, MD, USA) for bacterial counting every 2 h for 48 h. The experiment was repeated three times. In parallel, the same experiments were set to measure the OD_600_ values every 2 h for 48 h by using a fast automatic growth curve analyzer (OyGrowth Curves Ab Ltd, Finland). For both SA14^+^ and SA14, eight repeated samples were measured at each time point.

### Serum bactericidal test, cell adhesion and invasion assay, and anti-phagocytosis test

Bacterial capacity of anti-serum bactericidal activity was evaluated using both mouse and pig sera. Briefly, 25 µL bacterial culture of SA14^+^ and SA14 at mid-log phase were added into 75 µL mouse or pig serum or inactivated mouse or pig serum (inactivated at 56°C for 30 min). The bacteria-serum mixture was then incubated at 37°C for 2 h, followed by a series of 10-fold dilutions being performed for bacterial counting. In the control experiment the same volume of PBS replaced the serum. The whole experiment was triplicated.

A previously reported methodology [16] was followed to facilitate the analyses of adhesion and invasion assays, HEp-2 cells (ATCC® CCL-23; approximately 10^6^ cells per well) in each well of a 6-well plate were infected with MRSA strains SA14^+^ or SA14 at mid-log phase to reach a multiplicity of infection (MOI) of 10:1 (bacteria: cells) and were incubated at 37°C for 90 min. After being washed using PBS for three times, cells were lysed in 1 ml of sterile distilled water. The appropriate diluted lysates were plated on TSA plates to count the adherent and intracellular bacteria. HEp‐2 cells were also incubated with lysostaphin (6 U) for 90 min before lysis. Plating was performed to count the intracellular bacteria alone. All experiments were performed thrice.

Anti-phagocytosis assay was also performed in accordance with previously described methods [16]. Briefly, MRSA strains (SA14^+^ or SA14) at log‐phase were used to infect RAW264.7 monolayers in each well of a 6-well plate to reach a MOI of 10:1. After incubations of the cells at 37°C for 90 min, extracellular bacteria in each well of the plate were killed by addition of lysostaphin (6 U) with an incubation at 37°C for 20 min. Following this, EDTA (50 µM) was added to end the reaction. Afterward, cells in each well of the plates were lysed by adding 500 µl of trypsin and appropriate diluted lysates were plated on TSA plates to count the intracellular bacteria. The experiments were repeated thrice.

### Biofilm formation

Biofilm assays were performed as described previously [17]. Overnight cultures of MRSA strains were diluted (1:100 *v*/*v*) and 200 µL each of the dilutes poured into the wells of a 6-well microtiter plate (Corning, Corning, NY). The plate was then incubated at 37°C for 24 h, and the bacterial culture was removed. The wells were washed once using PBS and the sessile bacteria were fixed using 200 µL formaldehyde for 30 min at 37°C. Afterward, the wells were washed once again using PBS and were stained using 200 µL 1% crystal violet for 15 min. Unbound crystal violet dye in each of the wells was removed and was washed out using PBS. Thereafter, the wells were dried for 2 hr at 37°C, and 100 μl of 95% ethanol was added to each well. Absorbance was recorded at 590 nm using a plate reader (BioTek *Synergy HT*, USA). The wells with a sterile medium were used as negative controls. The experiments were repeated at least thrice.

### Animal tests

Animal tests were performed at Laboratory Animal Center, Huazhong Agricultural University (Wuhan, China), and were approved by the University Research Ethics Committee with an ID of HZAUMO-2020-0056. All experimental animals were housed under the same atmosphere and were carried out under the guidelines established by the China Regulations for the Administration of Affairs Concerning Experimental Animals (1988) and Regulations for the Administration of Affairs Concerning Experimental Animals in Hubei province (2005).

In *Galleria mellonella* models, each of the 0.4 ~ 0.5 g *Galleria mellonella* larvae received intraperitoneal challenges with MRSA strains at 10^5^ CFU, 10^6^ CFU, 10^7^ CFU, and/or 10^8^ CFU. Control groups included larvae with challenges of PBS and those without any treatment. The number of survival larvae was recorded every 12 h until 144 h. In mouse models, 5-week-old BALB/c mice were divided into 16 groups and each group contained 6 mice. Each mouse in different groups was intraperitoneally challenged with MRSA strains at different doses of PBS or received no treatment (Table 2). After challenge, the mortality was observed and the minimum lethal dose (MLD) was calculated. At 6, 12, 24, 48 hours post challenge mice bloods were harvested and the production of IL-8, IFN-γ, and TNF-α was detected using commercial cytokines detection kits (Dakewe Biotech, Shenzhen, China). At 7 days post challenge, surviving mice in each group were euthanized, and main organs (lungs, livers, and spleens) were collected for histological examination.

**Table 2.** Minimum lethal dose (MLD) of *S. aureus* SA14 and SA14^+^ in mouse models

Strain	Concentration (CFU)	Volume (μL)	Death/total	Death rate	MLD (CFU)
SA14	1.0 × 10^9^	200	6/6	100%	7.5 × 10^7^
	0.5 × 10^9^	200	6/6	100%	
	1.0 × 10^8^	200	6/6	100%	
	7.5 × 10^7^	200	2/6	33.3%	
	5.0 × 10^7^	200	0/6	0%	
	1.0 × 10^7^	200	0/6	0%	
	5.0 × 10^6^	200	0/6	0%	
	1.0 × 10^9^	200	6/6	100%	1.0 × 10^7^
SA14^+^	0.5 × 10^9^	200	6/6	100%	
	1.0 × 10^8^	200	6/6	100%	
	0.5 × 10^8^	200	6/6	100%	
	1.0 × 10^7^	200	4/6	66.6%	
	5.0 × 10^6^	200	0/6	0%	
	1.0 × 10^6^	200	0/6	0%	
PBS	–	200	0/6	0%	
Negative	–	200	0/6	0%	

### Illumina sequencing and Oxford Nanopore sequencing

The genomic DNA of phage PHB21 was extracted using the phenol-chloroform protocol [18]. After analysis through electrophoresis on a 1% agarose gel as well as a Qubit 2.0 (Thermo Scientific, Waltham, USA), a NEBNext Ultra^TM^ II DNA Library Prep Kit (NEB, Ipswich, USA) was used to prepare the sequence libraries, which were then sequenced on an Illumina NovaSeq 6000 platform (Novogene Co. LTD, Tianjin, China), using the pair-end 350 bp sequencing protocol. Raw reads with low quality were filtered as previously described [13,14]. High-quality reads were *de novo* assembled using SOAPdenovo2.04 with default parameters [19]. Terminal sequences of the phage genome were determined by a previously described modified statistical method [20]. Sequence annotation was performed using the RAST Serve [21]. Sequence alignments were performed and visualized using Easyfig v.2.0 [22]. Phylogenetic trees were generated using MEGAX with 1000 Bootstrap replications [23].

Oxford Nanopore sequencing (ONT) in combination with the Illumina sequencing was performed to generate the complete genome sequence of the MRSA lysogenic strain SA14^+^. Briefly, the genomic DNA of SA14^+^ was extracted using a commercial bacterial DNA preparing kit (TIANGEN, Beijing, China). DNA quality and quantity were evaluated as described above. Afterward, an ONT SQK-LSK109 Kit and a NEBNext® Ultra™ DNA Library Prep Kit were used to prepare DNA libraries for ONT sequencing and Illumina sequencing, respectively. Prepared DNA libraries were then sequenced using Nanopore PromethION platform and Illumina NovaSeq PE150, respectively. ONT and Illumina short reads were finally assembled and combined using the Unicycler v0.4.4 software with default parameters. Sequence was also annotated using the RAST Serve [21].

### RNA-Seq

To facilitate transcriptome analysis, MRSA strains SA14^+^ and SA14 were cultured in TSB to mid-log phase (OD_600_ = 0.65). Total RNAs were extracted from each of the samples using Trizol Reagent (Invitrogen, Carlsbad, CA, USA). The quality and quantity of the RNAs isolated were evaluated using NanoPhotometer spectrophotometer from IMPLEN (LA, CA, USA). Sequencing libraries were generated from the qualified RNA samples using a NEBNext® Ultra-RNA Library Prep Kit following manufacturers’ instructions. The libraries were then sequenced on an Illumina Hiseq 2000 platform at Novogene Co. Ltd (Beijing China) and 100-bp paired-end reads were generated. Raw reads obtained from the sequencing were processed using the in-house perl scripts. Next, clean reads with high quality was generated from the raw reads by removing reads containing adapter, reads containing ploy-N, and low-quality reads. Differential expressed genes (DEGs) were determined based on the clean data by using the DESeq R package (1.10.1) with p-value < 0.05 being set. Differential expressed genes were finally annotated by a GOseq R package for Gene Ontology (GO) analysis and a KOBAS software for KEGG analysis. GO terms with corrected p-value less than 0.05 were considered significantly enriched.

### RT-PCR assay

The transcription level of 16 virulence associated genes (*clpP1, cap8B, cap8A, cap8F, cap8D, cap8C, katA, esxA, cap8E, htpB, cap8G, clpC, clpP2, icaR, cpsK, brkB*) were also determined by RT-PCR. Primers are listed in Table S in supplementary materials. Total RNAs were extracted from SA14^+^ and SA14 as described in the *RNA-Seq* section. The relative transcription levels of the genes are shown as a ratio of the target gene to the reference gene using the formula *2^−(ΔΔCt)^* [24].

### Statistical analysis

Statistical analysis was performed through the “Two-way ANOVA” or “Multiple t tests” strategy in GraphPad Prism 8.0 (GraphPad Software, San Diego, CA). Data represents mean ± SD. The significance level was set at *P* < 0.05 (*).

## Results

### Isolation and characterization of a MRSA-specific temperate phage

**Figure 1.**
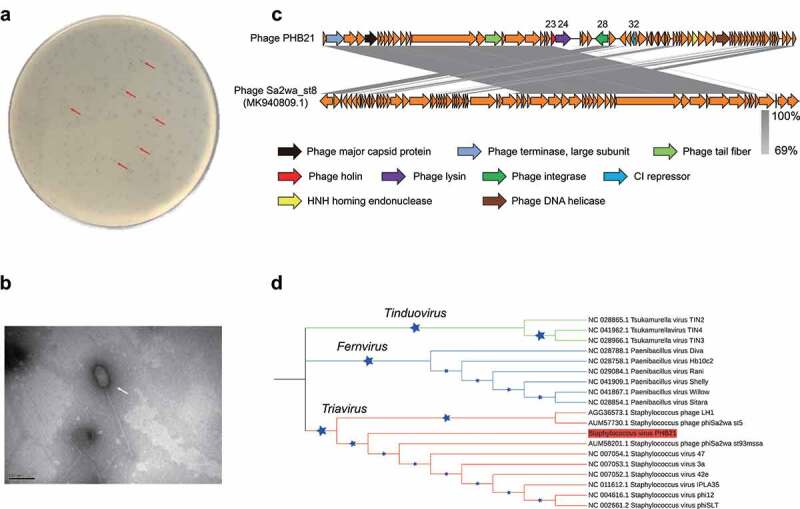
Characteristics of bacteriophage vB_Saus_PHB21. (a) Plaques (indicated using red arrows) formed by PHB21 on Methicillin-resistant *Staphylococcus aureus* growing agar; (b) morphological characteristics of phage PHB21 under the electron microscopy; (c) comparative analysis of PHB21 complete genome sequence and the complete genome sequence of *Staphylococcus* phage Sa2wa_st8 (GenBank accession no. MK940809.1); arrows refer to the coding sequence regions; (d) phylogenetic analysis of PHB21 and the other *Staphylococcus* phages; the tree was generated based on nucleotide sequences of the large terminase subunit encoding genes.

Using a MRSA strain SA14 as the indicator, we isolated a bacteriophage PHB21 from an epidermal sample collected from a Siberian tiger (*Panthera tigris altaica*) in Qingdao Zoo (Qingdao, China). Phenotypically, PHB21 formed small round translucent plaques with a clear boundary in the double agar (Figure 1(a)). Electron microscopy showed PHB21 particles had a prolate capsid (length 166 nm ± 3, width 69 nm ± 3) and a long flexible tail (312 nm ± 3) (Figure 1(b)). These morphological characteristics indicated that PHB21 belonged to the *Siphoviridae* family, according to the latest International Committee on Taxonomy of Viruses (ICTV) classification.

Illumina sequencing revealed that PHB21 possessed a linear double-stranded DNA genome with a size of approximately 45.26-Kb in length, with an average G + C content of 33.96%. BLASTn analysis revealed that the genome sequence of PHB21 was highly homologous (nucleotide identity 98.57%) to that of a prophage harbored in the genome of *S. aureus* strain NX-T55 (GenBank accession no. CP031839). Bioinformatical analyses showed that PHB21 genome did not contain any VFGs and it contained 72 genes encoding proteins involved in DNA packing and morphogenesis, lysis, replication, and regulation (Figure 1(c)). These proteins included a phage integrase (CDS 28) which was highly homologous to that of *Staphylococcus* phage Sa2wa_st8 (GenBank accession no. MK940809.1; Figure 1(c)), a CI repressor protein (CDS32) which was involved in phage lysogeny process, and an endolysin (CDS24) (Figure 1(c)). Phylogenetic analysis based on the nucleotide sequence of the large terminase subunit (ORF1) encoding genes showed that PHB21 was a member of the *Triavirus* species (Figure 1(d)).

We next performed spot tests to evaluate the lytic capacity of PHB21. A total of 30 MRSA strains of pig or human origin as well as eight strains belonging to the other bacteria species were included for analysis (Table 1). The results revealed that PHB21 had a lytic effect on 13 of the 30 MRSA tested; it displayed no lytic effects on the two *Staphylococcus chromogenes* isolates, *P. multocida, B. bronchiseptica, Salmonella, E. faecalis*, and *E. coli* (Table 1).

### Determination of phage integration site through ONT sequencing

**Figure 2.**
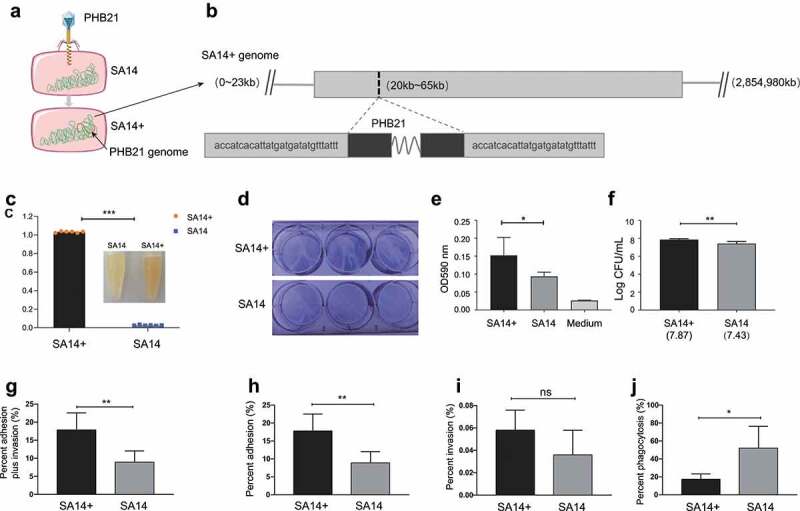
Generation and phenotypical characteristics of PHB21 lysogens. (a) A model for the generation of PHB21 lysogens; (b) the attachment site (attP) of PHB21 in the chromosome of lyse the lysogenic strain SA14^+^ determined using ONT sequencing; panel (c) indicates the staphyloxanthin produced by SA14^+^ and SA14 during the culture (at 24 h post inoculation); panels (d) to (f) show the biofilm formation of SA14^+^ and SA14 in crystal violet staining (panel D), OD_590_ measurement (panel (e)), and bacterial counting (panel (f)); panels (g) to (j) show the capacities of SA14^+^ and SA14 adherence and invasion to host cells (panel (g), percent adhesion plus invasion [*P* = 0.008]; panel (h), percent adhesion [*P* = 0.008]; panel (i), percent invasion [*P* = 0.120]), and anti-phagocytosis (panel (j), *P* = 0.014). Data represents mean ± SD and were analyzed through “Multiple t tests”. The significance level was set at *P* < 0.05 (*) or *P* < 0.01 (**). Raw data for cell adhesion, invasion, and anti-phagocytosis assays are shown in Table S3 in supplementary materials.

To determine the integration site of PHB21 genome, we first used this phage to infect the host bacterial strain SA14, and screened the lysogenic strain SA14^+^ (Figure 2(a,b); Figures S1A&B in supplementary materials). Through Oxford Nanopore Sequencing (ONT), we generated the complete genome sequence of SA14^+^ and the attachment site (attP) of PHB21 genome was determined. We found the attachment site of PHB21 genome was a 29-bp nucleotide sequence “ACCATCACATTATGATGATATGTTTATTT” (Figure 2(b)). In addition, the phage genome was integrated into a putative coding sequence region (CDS28) of SA14 and led to the CDS disruption. Annotation through different tools suggested that this coding sequence region encoded a hypothetic protein.

### Effect of phage-integration on the biological characteristics of MRSA

To explore the influence of the integration of PHB21 genome on the biological characteristics of MRSA strain SA14, we performed a series of laboratory tests. First, we observed the morphologies of both SA14^+^ and SA14, and we did not find any observed differences on bacterial morphology and size between the two strains under transmission electron microscope (Figures S1C&D in supplementary materials). We then measured the bacterial growth curve and tested the bacterial tolerance against mouse and swine sera, and we found SA14^+^ and SA14 showed similar growth curve (Figures S1E&F in supplementary materials) and capacity of anti-serum bactericidal effects (Figures S1G&H in supplementary materials). However, the lysogenic strain SA14^+^ secreted more staphyloxanthin than the parent strain SA14 during the culture (Figure 2(c)). Compared to SA14, the lysogenic strain SA14^+^ showed increased capacities of biofilm formation (Figure 2(d-f)), cell adhesion (Figure 2(g,h); Table S3 in supplementary materials), and anti-phagocytosis (Figure 2(j); Table S3 in supplementary materials). However interestingly, there was no significant difference on the cell invasion of SA14^+^ and SA14 (Figure 2(i); Table S3 in supplementary materials).

**Figure 3.**
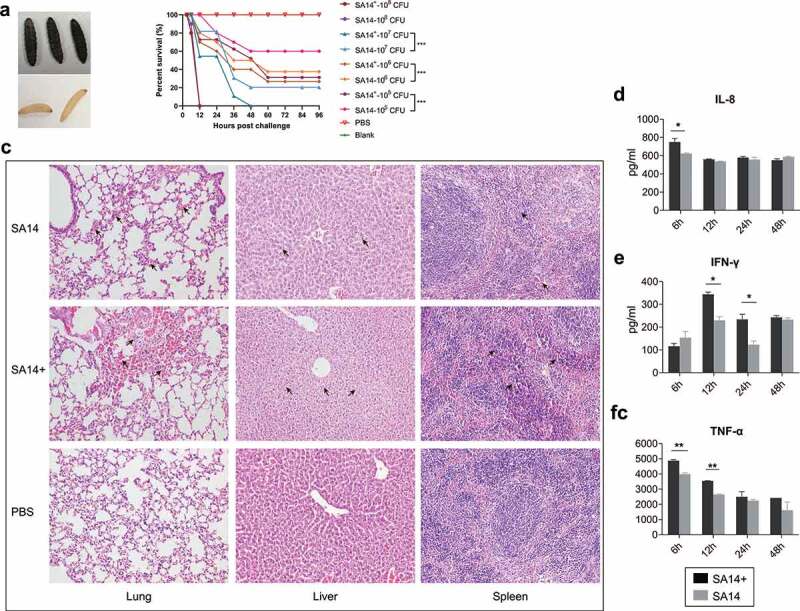
Evaluation of bacterial virulence of MRSA strains SA14^+^ and SA14 using different models. Panel (a) shows the color change of *Galleria mellonella* larvae challenged by MRSA strains; when a larva died due to bacterial infection its color turned to black; panel (b) displays the mortality curves of *Galleria mellonella* larvae due to the infections caused by different concentrations of MRSA strain SA14 and SA14^+^; panel (c) exhibits the histological damages (indicated by black arrows) on mouse lungs (HE, ×200), livers (HE, ×200), and spleens (HE, ×200) due to the infections of different MRSA strains; panels (d) to (f) show the production of different cytokines (IL-8, IFN-γ, and TNF-α) in mouse sera induced by the infections of different MRSA strains at different time points post challenge. Data represents mean ± SD. The significance level was set at *P* < 0.05 (*).

We next evaluated and compared the virulence of SA14^+^ and SA14 using both *Galleria mellonella* and mouse models. In *Galleria mellonella* models, larvae of *Galleria mellonella* (0.4 ~ 0.5 g per larva, 10 larvae in each of the groups) were challenged with different doses (10^5^ CFU, 10^6^ CFU, 10^7^ CFU, and/or 10^8^ CFU per larva) of SA14^+^ and/or SA14. The number of survival larvae was recorded every 12 h until 144 h. The results revealed that challenge of SA14^+^ induced more severe mortality than SA14 at the same dose (Figure 3(a,b)). As measured in mouse models, the minimum lethal dose (MLD) of SA14^+^ to mice (BALB/c, 5-week-old) was 1.0 × 10^7^ CFU, which was approximately 7.5-fold lower than that of SA14 (7.5 × 10^7^ CFU) (Table 2). Histologically, mice challenged with SA14^+^ showed more severe lesions in lung, liver and spleen compared to those challenged with SA14 (Figure 3(c)). While challenge of SA14 and SA14^+^ caused hemorrhage in the lungs of mice compared to those of the mice treated with PBS, more inflammatory exudates mixed with red blood cells were observed in the alveoli of the lungs of mice challenged with SA14^+^. The livers of mice challenged with SA14^+^ showed disturbed hepatic cord arrangement, narrowed sinusoidal space, swelling of hepatocytes, cytoplasmic laxity of hepatocytes with light staining and vacuolization, which were more severe than those in the livers of mice challenged with SA14 and PBS. In the spleens of mice challenge with SA14^+^ and SA14, the red pulp is enlarged and filled with blood compare to the spleens of mice treated with PBS, but a more severe pathological change was observed in the spleens of SA14^+^-infected mice. In particular, challenge of SA14^+^ induced higher levels of IL-8, IFN-γ, and TNF-α in mice (Figure 3(d-f)).

### Revealing the mechanism of phage enhancing the virulence of MRSA through RNA-Seq

**Figure 4.**
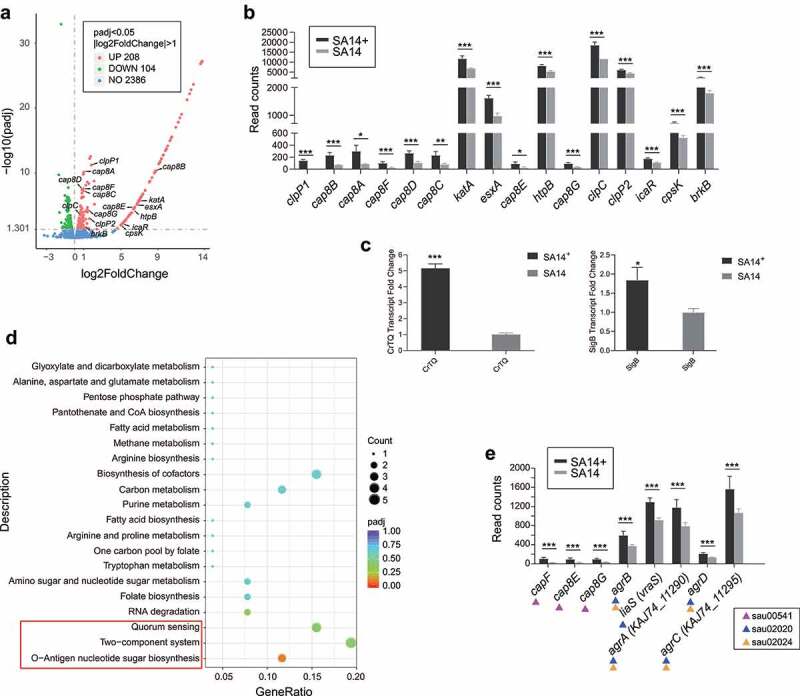
Transcriptome analysis of the MRSA lysogenic strain SA14^+^. (a) Scatter plot displays the differentially expressed genes in the lysogenic strain SA14^+^ compared to those in the wild type strain SA14; (b) column chart shows the transcription of virulence associated genes in the lysogenic strain SA14^+^ compared to those in the wild type strain SA14; (c) column chart shows the transcription of with the synthesis and export of staphyloxanthin in the lysogenic strain SA14^+^ compared to those in the wild type strain SA14; (d) enriched KEGG pathways related to the upregulated genes expressed in SA14^+^ compared to the wild type strain SA14; (e) the transcription of genes related to O-antigen nucleotide sugar biosynthesis (KEGGID: sau00541), two-component system (KEGGID: sau02020), and quorum sensing (KEGGID: sau02024) in MRSA strains SA14^+^ and SA14. Data represents mean ± SD. The significance level was set at *P* < 0.05 (*).

To answer the question why the integration of the non-VFG-carrying phage PHB21 enhances the virulence of the host bacterium, total of RNAs extracted from both SA14^+^ and SA14 at the same time point were sent for transcriptome sequencing. This strategy identified a total of 312 differentially expressed genes (DEGs) in SA14^+^ compared to SA14, including 208 significantly upregulated DEGs and 104 downregulated DEGs (|log_2_(FoldChange)| > 1, *p*_adj_ < 0.05; Figure 4(a); Table S2 in supplementary materials). In particular, a total of 16 genes associated with the virulence of *S. aureus* exhibited upregulated expressions the lysogenic strain (*clpP1, cap8A, cap8B, cap8C, cap8D, cap8E, cap8F, cap8G, katA, esxA, htpB, clpP2, icaR, cpsK, brkB*, and *clpC*) (Figure 4(b)). The transcription profiles of these genes were verified by separate RT-PCR assays (Supplementary materials Figure S2). In particular, genes associated with the synthesis and export of staphyloxanthin showed up-regulated transcriptions in SA14^+^ compared to those in SA14 (Figure 4(c)).

Kyoto Encyclopedia of Genes and Genomes (KEGG) analysis revealed that the genes upregulated expressed in the lysogenic strain were enriched to several pathways beneficial for bacterial fitness and pathogenesis (Figure 4(d,e)). Of particular note are the O-Antigen nucleotide sugar biosynthesis pathway (KEGGID: sau00541), the two-component system pathway (KEGGID: sau02020), and the quorum sensing (KEGGID: sau02024) pathway, because the upregulation of these three pathways have been shown to have positive impacts on bacterial biofilm formation and virulence [17,25–27].

## Discussion

The effect of phages on bacteria is like a “double-edged sword”: phages can either kill bacteria or they can enhance the bacterial fitness and virulence through different ways [28]. Many phages in particular temperate phages have been found to carry VFGs (such as toxin-encoding genes) and can spread these harmful genes to bacteria through integration, thereby enhancing bacterial virulence [11,12]. In this study, the phage PHB21 was isolated from the epidermal sample of a Siberian tiger using a human origin MRSA strain SA14 as the indicator. Whole genome sequencing and bioinformatical analysis revealed that PHB21 genome encoded a phage integrase (CDS 28), and a CI repressor protein (CDS32). The presence of these two genes suggests that PHB21 is a temperate lysogenic phage, since phage integrases mediate unidirectional site-specific recombination between two DNA recognition sequences [29], while CI represses phage lytic genes and commits the phage to the lysogenic program [30]. Expectedly, we found the genome of PHB21 could be integrated into the chromosome of the host MRSA strain SA14 through a specific attachment site and the lysogenic strain SA14^+^ was also obtained. However, PHB21 still displayed a capacity to kill several MRSA strains. This might be because the genomes of these MRSA strains lack the matched attachment sites, and the phage maintains the lytic cycle to those strains [10]. In addition, those bacteria that could not be killed by PHB21 might be because they contain prophages similar to the phage, and these prophages mediate defense against PHB21 infection [31].

Through ONT sequencing, we found that PHB21 genome was integrated into a coding sequence encoding a hypothetic protein. It seems that the disruption of this gene has no effects on the bacterial morphology, growth, and serum-resistance, as we did not see observed differences on these phenotypes of the lysogenic strain SA14^+^ compared to the wild type strain SA14. However, the lysogenic strain SA14^+^ showed increased yellow/orange in color and biofilm formation. These findings are similar to those from another study [17], and a proposed reason might partly explain the findings is the actions of phage CI protein induce an activation of the alternative sigma factor (SigB) regulon, which provokes an increased secretion of an important virulence factor staphyloxanthin and biofilm formation [17,27]. An increased staphyloxanthin secretion could be indicated by a more intense yellow/orange color of the phage-integration strain during the culture [17]. In addition, an increased staphyloxanthin secretion and biofilm formation might also indicate an increased virulence since both of them positively impact the pathogenesis and resistance of MRSA [32]. In addition, bacterial adhesion is also a key defining feature of biofilm, and adhesion is also recognized as a weapon in microbial competition [33]. In agreement with these findings, the lysogenic strain SA14^+^ displayed increased cell adhesion, anti-phagocytosis, and virulence to both *Galleria mellonella* and mice compared to those of the host strain SA14. In addition, infection of both SA14^+^ and SA14 induced the production of IL-8, IFN-γ, and TNF-α. These results are also in agreement with the other studies [34,35]. However, infection of SA14^+^ induced higher levels of IL-8, IFN-γ, and TNF-α than the infection of SA14 in the early stage, but there was no significant difference in the late stage. This might be because SA14^+^ had a stronger capacity of forming biofilm than SA14, and *S. aureus* biofilms can attenuate the host inflammation [35].

Our transcriptional analysis determined a number of genes differentially transcripted in the lysogenic strain SA14^+^ compared to those in the wild type strain SA14. Of particular note were 16 genes which are important in the pathogenesis of *S. aureus*. For example, *cap8A, cap8B, cap8C, cap8D, cap8E, cap8F*, and *cap8G* involve in the biosynthesis of capsule, which contributes to the virulence and antiphagocytosis of *S. aureus* [36–38]; *clpP1, clpP2,* and *clpC* participate in the encoding of Clp chaperones, ATPases and proteases; both of these proteins are central in stress survival, virulence and biofilm formation of *S. aureus* [39,40]; the product of *katA* (catalase enzyme KatA) has a capacity to decompose hydrogen peroxide, which is a reactive oxygen intermediate and is indispensable for the bactericidal activity of phagocytes [41]; *esxA* encodes the type VII secretion system (T7SS) effector protein EsxA which is pivotal for bacterial virulence [42]; the heat shock protein coding gene *htpB* encodes a 60-kDa chaperonin with an essential function as mediators of protein folding; this protein is important in bacterial pathogenesis and could mediate bacterial attachment to and invasion of host cells [43,44]; *icaR* is a known biofilm regulator gene in *Staphylococcus* and its upregulation contributes to the biofilm formation [45]; *cpsK* encodes the teichoic acids export ABC transporter permease subunit TagG which is beneficial for the export of bacterial polysaccharides-wall teichoic acids, thereby contributing to the polysaccharides production [46]. In addition, the upregulated-expressed genes induced by the integration of PHB21 genome in the lysogenic strain SA14^+^ are related to several KEGG pathways that have previously been reported to have important roles in the biofilm formation, stress tolerance, and pathogenesis of *Staphylococcus* [17,27]. The upregulated expression of these genes including the virulence associated genes in the lysogenic strain may explain why the lysogenic strain SA14^+^ displayed increased virulence, biofilm formation, cell adhesion, and anti-phagocytosis compared to the wild type strain SA14.

To be concluded, we isolated a temperate MRSA *Siphoviridae* bacteriophage from an epidermal sample of Siberian tiger (*Panthera tigris altaica*) in this study. Although this temperate phage did not carry any virulence genes and still exhibited bactericidal activities to several MRSA strains, it was found to be able to improve the biofilm formation, stress tolerance, and virulence of the lysogenic MRSA strain. This striking phenomenon could be explained through the following reason in mechanism: the integration of the phage genome into the bacterial chromosome led to the upregulated expression of many genes related to the virulence and biofilm formation, which thereby conferring the corresponding phenotypes to the lysogenic strain. Our results presented herein may provide novel knowledge of “bacteria-phage-interactions” in MRSA. This study may also remind a cautious way to set phage-therapy for MRSA infections; the safety of a phage intends to be used should be fully evaluated.

## Supplementary Material

Supplemental Material

## Data Availability

All sequence data generated in this study are deposited into NCBI database under the Bioproject (accession number: PRJNA720778). GenBank accession numbers for the complete genome sequences of *Staphylococcus aureus* bacteriophage phage vB_Saus_PHB21 and *Staphylococcus aureus* lysogenic strain SA14^+^ are MW924497 and CP073012, respectively. Sequence Read Archive (SRA) accession number for the transcriptome sequence is SRR14193019.
